# The Management of Cardiovascular Risk through Epigenetic Biomarkers

**DOI:** 10.1155/2017/9158572

**Published:** 2017-07-13

**Authors:** Laurent Metzinger, Stefano de Franciscis, Raffaele Serra

**Affiliations:** ^1^CURS, Laboratoire INSERM U1088, Université de Picardie Jules Verne, chemin du Thil, 80025 Amiens Cedex 1, France; ^2^Interuniversity Center of Phlebolymphology (CIFL), International Research and Educational Program in Clinical and Experimental Biotechnology, University Magna Graecia of Catanzaro, Viale Europa, 88100 Catanzaro, Italy; ^3^Department of Medical and Surgical Sciences, University Magna Graecia of Catanzaro, Viale Europa, 88100 Catanzaro, Italy

## Abstract

Epigenetic sciences study heritable changes in gene expression not related to changes in the genomic DNA sequence. The most important epigenetic mechanisms are DNA methylation, posttranslational histone modification, and gene regulation by noncoding RNAs, such as microRNAs (miRNAs) and long noncoding RNAs (lncRNAs). Cardiovascular diseases (CVD) are responsible for at least one-third of premature deaths worldwide and represent a heavy burden of healthcare expenditure. We will discuss in this review the most recent findings dealing with epigenetic alterations linked to cardiovascular physiopathology in patients. A particular focus will be put on the way these changes can be translated in the clinic, to develop innovative and groundbreaking biomarkers in CVD field.

## 1. Introduction

Epigenetics is the study of all the heritable changes in gene expression that do not involve changes to genomic DNA sequences in themselves. Epigenetic mechanisms represent a stable cellular memory that allows the propagation of gene activities from one generation of cells to another. The three main epigenetic processes are represented by DNA methylation, posttranslational histone modifications, and RNA-based mechanisms such as noncoding RNAs, represented mainly by microRNAs (miRNAs), and long noncoding RNAs (lncRNAs) [1, 2].

Cardiovascular diseases (CVD) are responsible for one-third of all deaths worldwide and accounting for an important burden of healthcare expenditure. Previously, epigenetic modifications were reported to play a pivotal role in processes underlying CVD, including atherosclerosis, inflammation, and hypertension [3]. To date, most of the limitations for the full understanding of the genetic influence on cardiovascular diseases (CVD) are probably due to the static simple evaluation of the DNA code. In this context, epigenetics, through the study of several dynamic pathways, modify also the genome's functionality under exogenous influence, which could identify novel mechanisms and targets in the control of gene regulation, with significant acquisitions in CVD knowledge of its genetic risk and pathophysiology [1–3]. Indeed, epigenetic modifications such as histones modifications, DNA methylation, and small noncoding RNAs occur in response to environmental changes. Pollution and diet will profoundly change these epigenetic modifications and trigger susceptibility to CVD. For example, as will also be discussed in this review, DNA methylation has been associated with atherosclerosis [4], abdominal aortic aneurysm [5], and coronary heart disease [6].

This review will thus focus on the role of the main mechanisms of epigenetics in the area of cardiovascular risk.

## 2. Literature Search

In the context of CVD, we decided to search for relevant articles in the three main areas of interest of epigenetics: DNA methylation, posttranslational histone modifications, and RNA-based mechanisms. PubMed, Scopus, and Science Direct databases were used for the search strategy. A PubMed search using the terms cardiovascular + epigenetic + biomarker found 261 results in May 2017.

## 3. DNA Methylation

DNA methylation, partly controlled by DNA methyltransferases, is usually associated with transcriptional repression, while demethylation is associated with transcriptional activation, influencing gene expression by altering DNA promoter accessibility to RNA polymerase, and consequently gene transcription [7]. A PubMed search using the terms cardiovascular + methylation + biomarker + biomarker found 185 results in May 2017.

In this context, the concept of global methylation refers to the overall level of methylcytosines in the genome, expressed as percentage of total cytosines. In fact, a large proportion of methylation sites within the genome are found in repeat sequences and transposable elements, such as Alu and long-interspersed nuclear element (LINE-1) and this correlates with the total genomic methylation content, and, for this reason, LINE-1 is used as a surrogate for the overall methylation of the genome [3]. Interestingly, LINE-1 methylation is increased in males [21], while it is not affected by either age or natural cycle of hormones.

Several research teams have studied the possibility that DNA methylation could be correlated with the risk of CVD. Several evidences showed that global DNA methylation assessed at LINE-1 sequences was inversely and independently related to CVD risk; thus more proportions of global DNA methylation are indicative of a higher CVD risk [3, 22, 23]. Very recently, a Swedish team performed an epigenome-wide association study to identify disease-specific alterations in DNA methylation in blood samples of a Swedish population of 729 patients, afflicted with hypertension, myocardial infarction, stroke, thrombosis, and cardiac arrhythmia [24].

For this, they used an Illumina Infinium BeadChip. Differential DNA methylation was detected in more than 200 CpG-sites in patients with a history of myocardial infarction. Among these sites, 42 genes were related to cardiac function. The authors concluded that individuals with a history of MI have an altered pattern of DNA methylation at numerous genomic loci linked to CVD. These sites are thus potential biomarkers for CVD [24]. DNA methylation in specific genes can also be helpful to predict response to a specific treatment. Two elegant studies by Gallego-Fabrega et al. have shown that changes in DNA methylation patterns of PPM1A and TRAF3 are, respectively, associated with vascular recurrence in aspirin-treated patients and clopidogrel response and recurrence of ischemic events in patients with stroke [25, 26].

## 4. Posttranslational Histone Modifications

Eukaryotic DNA is wrapped around an octamer of the core histones that build the fundamental unit of chromatin, the nucleosome. These chromatin elements are unstable and they change rapidly in response to any external stimuli, and any permanent changes to DNA can lead to the development of defective organs or the development of a disease [1]. Very sparse studies tend to show that histone modifications are linked with CVD. In normal mice fed with propylthiouracil (PTU, an inhibitor of T3 production), Pandya et al. have shown that the distribution of histone 3 lysine 4 trimethylation at a myosin-heavy chain related locus was reversibly altered in ventricles, suggesting that histone 3 lysine 4 trimethylation modification is an epigenetic marker associated with changes in myosin-heavy chain gene regulation [27]. Histone acetylation is a dynamic process regulated by histone acetyltransferases and histone deacetylases (HDACs). The balance between these two enzyme families is crucial to regulating gene expression and could be incriminated in CVD development [28]. In rats, inhibiting a specific type of HDAC in the heart promotes cardiac stem cell-promoted cardiac regeneration which in turn induces a partial restoration of cardiac function [29]. These animal studies give hope that histone modification is a promising way to develop innovative biomarkers and therapies in the CVD field.

To date, there are however very few significant reports in human related to posttranslational histone modifications in the area of CVD risk to be discussed. Ek et al., using a genomewide DNA methylation study, have shown that growth-differentiation factor 15 is associated with myocardial infarction and that, interestingly, growth-differentiation factor 15 mRNA was regulated by a specific small RNA, miR-21, leading us to the next chapter in our review [30].

## 5. RNA-Based Mechanisms

### 5.1. Noncoding RNAs in the Management of Cardiovascular Risk

Noncoding RNAs including microRNAs (miRNAs) and the recently discovered long noncoding RNAs (lncRNAs) are defined as a novel class of endogenous RNAs that regulate the human genome and are not translated into proteins. They have been shown to be implicated in cardiovascular physiopathology. A large number of long noncoding RNAs and microRNAs were in recent years implicated in cellular and animal models of cardiovascular diseases (CVD) and were shown to be deregulated in patients with CVD. We will discuss here their potential role as biomarkers in the CVD context. Also, as the miRNAs and lncRNAs we discuss may represent novel targets to treat or prevent CVD, we will see how they could be used for the delivery of new treatments in CVD using groundbreaking techniques.

### 5.2. miRNAs as Biomarkers in CVD

It has been now 17 years since the existence of microRNAs (miRNAs) was shown in human. These short endogenous interfering RNAs are coded by the human genome, represent a new class of thousands of RNAs of approximately 20 to 25 nucleotides (see specialized Internet databases, such as miRbase http://www.mirbase.org/cgi-bin/mirna_summary.pl?org=hsa), and have emerged as regulators of numerous physiological and pathological processes [31], including CVDs [32, 33]. Their number is now estimated to be around 2,000 but a recent study using thorough RNA purification from human cells and next generation sequencing claims that there exist up to 5,000 miRNAs, with more than a half being human specific [34].

### 5.3. miRNA Biogenesis

In the canonical pathway, a large RNA precursor, pri-miRNA, is transcribed and matured in the nucleus by the RNA polymerase II. It is subsequently cleaved near the hairpin stem base, by RNase III Drosha and protein partner, Di George syndrome critical region 8. Thus the pre-miRNA precursor, a hairpin of approx. 60 to 70 nucleotides, is obtained which is then released in the cytoplasm. It will be cleaved in its stems by the RNase III Dicer. This results in the double stranded miRNA/miRNA^*∗*^ duplex (21–25 base-pairs) which is unwound by RNA-induced-silencing complex (RISC). RISC will then carry the mature miRNA to target messenger RNAs resulting in gene silencing. Most of the time the binding will take place in the 3′ untranslated region and will decrease target mRNA levels and translation [35]. Noncanonical pathways such as the mirtron pathway (in this instance miRNAs arise from introns) have also been described [36].

### 5.4. MicroRNAs as Predictors of CVD Outcome

As is abundantly explained in this special issue, CVD are one of most common causes of premature death in the general population, with an increasing number of individuals at risk. New biomarkers are needed to better stratify the risk of progression or the risk of specific complications associated with CVD, and noncoding RNAs are prime candidates. A PubMed search using the terms cardiovascular + mirna + biomarker brings 969 results in May 2017, further highlighting the interest in the field.

It was reasonable to hypothesize that miRNAs would be differentially expressed between the general population and patients at risk of developing CVD or suffering from CVD. For instance, we have shown that several miRNAs are differentially expressed in the carotid plaque between symptomatic patients (having developed a stroke of ischemic transient episode) and asymptomatic patients (having carotid plaques removed by surgery but without pathological episode) [37]. Often however the clinician does not have access to the pathological tissue. In a groundbreaking work, Mitchell et al. [38] have demonstrated that circulating miRNAs are present in the human blood, where they are carried in complex with the chaperon argonaute 2 and/or lipoproteins or by microvesicles, conferring stability and protection against RNases [39]. Correlations exist between blood miRNA levels and pathologies, indicating that miRNAs are potential noninvasive biomarkers [40]. It is thus reasonable to hypothesize that miRNA seric or plasma expression is a most promising avenue to evaluate early risk and patient outcome, but also individual patient response to various treatments or surgical procedures. At first, miRNAs were considered tissue and cell specific, and it was thus expected that their serum expression would reflect pathophysiology of concerned organs. This is now controversial and most miRNAs are rather enriched for a peculiar tissue. miRNAs are usually quantified using reverse transcription-quantitative PCR, so the appropriate internal control gene is very important for obtaining accurate results of miRNA expression. However, there still has been controversy about the choice of gene used as an internal control [41]. Currently, the most widely used and accepted internal controls for miRNA qPCR are spiked-in exogenous nonhuman miRNA (e.g., synthetic* Caenorhabditis elegans* miR-39), to avoid further experimental bias [41]. Please note that urinary miRNA also represents a potential novel source to discover noninvasive biomarkers for CVD [42].

We now know that cardiospecific miRNAs are highly stable in the blood [37]. The pioneering work of Dimmeler et al. highlighted a change in miRNA levels in the serum of patients with coronary artery disease (CAD) compared to controls [43], and miRNAs are now potential biomarkers for CVD [32]. Since this pioneering study, others have shown that miRNAs are biomarkers able to predict CVD outcome. For example, Seronde et al. have shown, in a cohort of 340 patients, that decreased seric levels of miR-423-5p were associated with a poor long-term outcome in acute heart failure patients [8], indicating the value of this miRNA as a prognostic biomarker of acute heart failure. The largest cohort published to date (1114 patients) indicates that seric levels of miR-132, miR-140-3p, and miR-210 were able to precisely predict cardiovascular death (the main outcome) in patients suffering from acute coronary syndrome diagnosis [9].

miRNAs are interesting biomarkers to predict CVD outcome in populations that are particularly at risk. This is, for instance, true in the chronic kidney disease (CKD) population that is especially at risk for CVD [10, 11]. Our experimental data [12, 13] suggests that miR-126, miR-143, miR-145, miR-155, and miR-223 are potential circulating biomarkers for the diagnosis and prognosis of patients with CKD to assess the levels of miRNA expression at various stages of CKD, such as vascular calcifications [13], justifying further studies to correlate alterations of miRNA levels with CVD outcome in CKD patients. Also the levels of several of these miRNAs are sensitive to circulatory stresses and could provide valuable data about the way patients regulate their blood pressure [14].

Gupta et al. have shown that miR-22 regulates cardiac autophagy, especially in the myocardium of senior patients, and that the circulating levels of miR-22 give prognostic information on eventual progression towards heart failure, giving interesting clues about miR-22's role as biomarker candidate for CVD in the elderly population [15]. miRNAs have also potential to predict the cardiovascular toxicity of drugs and could, for example, help to detect anthracycline-induced cardiac damage. Piegari et al. have shown that miR-34 can predict the cardiotoxic effects of the anticancer drug doxorubicin, at least in a murine model [16].

As miRNAs are potential biomarkers in type 2 diabetes mellitus, obesity, and cardiovascular diseases, they have also been proposed to monitor the physiological effects of exercise in the general population [44]. For example, miR-146a and miR-221 levels were decreased and miR-149^*∗*^ was increased after acute exercise [17], whereas endurance exercise altered the expression of miR-1, miR-133, and miR-206 [19]. The same was also true for patients at risk, such as a population of chronic kidney disease patients where acute exercise increased miR-150 levels and decreased miR-146a levels [18]. miR-1 was also described as a potential biomarker in an elderly population submitted to eccentric and conventional strength training [20].

Table 1 resumes the main miRNAs implicated in CVD.

### 5.5. Long Noncoding RNAs (lncRNAs)

We now know that miRNAs are regulated at least in part by lncRNAs, long transcripts (>200 nt) that do not code for proteins. The biogenesis and molecular mechanism of action of miRNAs and lncRNAs have been described in greater detail in other reviews [26, 33, 44, 45]. According to recent databases 56018 and 46475 lncRNA genes have been, respectively, found for human and mouse. lncRNAs differ from miRNAs, as they regulate gene expression not only at the transcriptional and but also at the posttranscriptional and chromatin remodeling levels. They also have high tissular specificity and are not evolutionarily conserved. lncRNA can act as decoy for microRNA to allow microRNAs' target mRNA to escape from degradation (reviewed by [44]). The use of lncRNAs as innovative biomarkers in the field is still in its infancy compared to the miRNA field. Some really promising candidates, such as LIPCAR [46] or UCA1 [47], have been published in the CVD field, and more are yet to come.

### 5.6. Gene Therapy via MicroRNAs: Can It Work in the CVD Field?

As miRNAs are promising biomarkers, they are also logical candidates to design innovative gene therapies, especially as several genes are involved in most cardiovascular disorders [48]. And miRNAs are master gene regulators able to regulate up to 100 mRNA targets, often implicated in convergent pathways [33]. We will however have to keep in mind several potential problems, such as loss of the therapeutic RNAs due to nucleases and/or uptake by macrophages and inefficient endocytosis by target cells.

Several successes have been accomplished in human patients, using antisense based miRNA inhibitors such as miravirsen, with minimal side effects [49]. On the other side, we can also use overexpression of miRNAs depending on the tissue and clinical context. Chad Mirkin's team has developed nanotechnologies based on gold nanoparticles that can be covalently linked with mature miRNA duplexes or antisense sequences. Interestingly, in rodents, they can cross the blood-brain/blood-tumor barriers when administered intravenously [50, 51]. miRNA-based therapies are at present mostly developed in the cancer field, but the CVD field will follow without doubt, with exciting times ahead of us.

## 6. Conclusion

Epigenetics is a relatively new science that has a tremendous potential to introduce new biomarkers in the CVD field and also new avenues for innovative therapies. There are several potential benefits of the use of innovative epigenetic biomarkers such as DNA methylation of specific genes (i.e., TRAF3 or PPM1A methylation patterns as explained in this review) or miRNAs (i.e., miR-132, miR-140-3p, and miR-210 as predictors of cardiovascular death). Compared to classic biochemical biomarkers, they can provide valuable data about gene functions and phenotypes that would be helpful concerning CVD diagnosis, outcome, prognosis, treatment monitoring, and stratification. However one has to keep in mind that, as with other scientific fields in their infancy, a significant number of clinical studies with large cohorts will now have to be implemented to confirm that interest. Also, biologists will have to harmonize the methods of detection in order to avoid biases linked to technical issues.

## Figures and Tables

**Table 1:** miRNAs implicated in CVD.

	Pathophysiology	Ref
miR-423	Acute heart failure	[8]
miR-132, -140, -210	Cardiovascular death	[9]
miR-126, miR-143, miR-145, miR-155, miR-223	Vascular calcifications linked to chronic kidney disorders	[10–14]
miR-22	Cardiac autophagy	[15]
miR-34	Cardiotoxic effect of doxorubicin	[16]
miR-146a, -150, miR-221, miR-149^*∗*^	Acute exercise	[17, 18]
miR-1, -133, -206	Endurance exercise	[19]
miR-1	Strength training	[20]
